# Case report: Surgical repair for left main coronary artery to right atrium fistula with endocarditis

**DOI:** 10.3389/fcvm.2023.1101750

**Published:** 2023-04-12

**Authors:** Weimin Zhang, Abdunabi Maimaitiaili, Yan Xing, Fei Yan, Qiang Huo

**Affiliations:** ^1^ Department of Cardiac Surgery, The First Affiliated Hospital of Xinjiang Medical University, Urumqi, China; ^2^ Imaging Center, The First Affiliated Hospital of Xinjiang Medical University, Urumqi, China

**Keywords:** coronary artery fistula, heart failure, echocardiography, computed tomography, surgery

## Abstract

Coronary artery fistula (CAF) is a rare coronary anomaly defined as a communication between coronary artery and other heart chambers or vascular structures. In this case report, a 32-year-old woman with a giant left main coronary artery to the right atrium fistula with endocarditis was presented. CAF was diagnosed by transthoracic echocardiography and subsequently confirmed by cardiac computerized tomographic and coronary angiography. The patient received antibiotic treatment for infective endocarditis for 6 weeks preoperatively. The fistula was successfully treated with surgical repair. The patient is well now after 18 months of follow-up.

## Introduction

Coronary artery fistula (CAF) is a rare coronary artery anomaly that originates from the coronary artery and drains into any cardiac chamber and great vessel. The estimated prevalence of CAF is about 0.002% in the general population and 0.2% in patients undergoing coronary angiography (1). Most CAFs originate from the right coronary artery (55%), the left anterior descending artery (35%), and both coronary arteries (5%) (2). The involvement of the left main coronary artery (LMCA) accounts for only 0.7% of CAFs (3). Endocarditis of CAFs in the cardiac chambers is extremely rare (4). Here, a case of a giant CAF connecting the LMCA to the right atrium (RA) with endocarditis was presented.

## Case report

**Figure 1:**
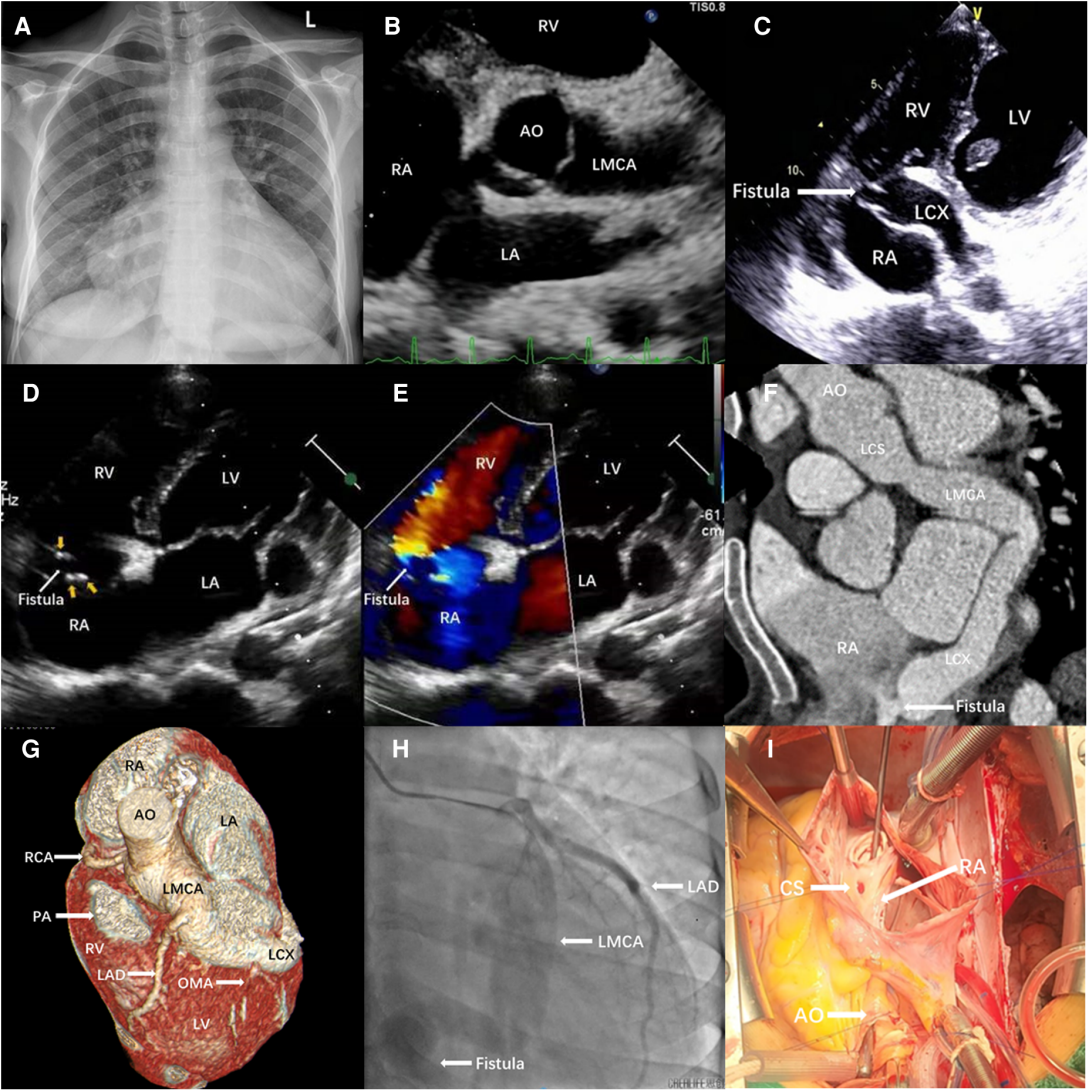
(**A**) Chest x-ray showed gross cardiomegaly. (**B–E**) Transthoracic echocardiography. (**B**) The parasternal short-axis image showed a large fistula, originating in LMCA. (**C**) Modified four-chamber view showed the fistula drained from LMCA into RA, adjacent to the coronary sinus. (**D**) A continuous systolic–diastolic flow inside the structure at color Doppler, which was confirmed at continuous wave Doppler directed from the LMCA toward RA. (**E**) Cardiac vegetations were found on the orifice of the fistula in the RA. Computed tomography angiography (**F,G**) and coronary angiography (**H**). A large fistula originating from LMCA and entering into RA was shown, and the LAD and OM arteries originate directly from LMCA (**G,H**). (**I**) Intraoperative findings. An abnormal vascular channel between left aortic sinus and RA (probe) was observed. AO, aorta; CS, coronary sinus; LA, left atrium; LAD, left anterior descending coronary artery; LMCA, left main coronary artery; LCX, left circumflex coronary artery; LV, left ventricle; OMA, oblique marginal artery; PA, pulmonary artery; RA, right atrium; RCA, right coronary artery; RV, right ventricle.

A 32-year-old Chinese woman presented with a 1-year history of chest tightness and chest pain after exertion and intermittent fever lasting for 1 month. Cardiac examination revealed continuous murmur at the right parasternal border. Laboratory findings were as follows: leucocytes, 10.15 × 109/L; hemoglobin, 86 g/L; C-reactive protein, 27.3 mg/L; IL-6, 31.56 pg/mL; erythrocyte sedimentation rate, 62 mm/h. Myocardial biomarkers were within normal range. Blood cultures taken at admission grew *Streptococcus sanguinis*. Electrocardiography displayed a sinus rhythm. Chest x-ray showed gross cardiomegaly (Figure 1A). Transthoracic echocardiography (TTE) showed that a giant fistula structure between the left coronary sinus (CS) and the RA, and the LMCA diameter was 19 mm (Figure 1B). The opening of the fistula in the RA was adjacent to the CS (Figure 1C), with a diameter of 6 mm. Blood initially passes from the high-pressure aorta to the RA, thereby leading to left-to-right shunt (Figure 1D). In addition, cardiac vegetations were found on the orifice of the fistula in the RA (Figure 1E). Few tiny calcified nodules were found at the aortic and mitral valves.

Cardiac computerized tomography showed obvious dilatation in the left CS and LMCA. LMCA originated from the left CS and coursed along the coronary sulcus to form the dilated left circumflex artery (LCX) (Figure 1F). The volume-rendered image demonstrates the fistulous communication to the LMCA and the RA (Figure 1G). Meanwhile, the left anterior descending artery (LAD) and the oblique marginal artery (OMA) originated from the LMCA. The proximal diameter of the LMCA was 20 mm. The LCX narrowed at the right atrial posterior wall and drained into the RA adjacent to the opening of the CS. Coronary angiography showed a very large CAF originating from the left CS and terminating directly into the RA (Figure 1H). LAD and OMA originated separately from LMCA, whereas the right coronary artery (RCA) was normal.

The patient received antibiotic treatment for infective endocarditis for 6 weeks in and out of hospital, as follows: Penicillin G (200,000 U/day i.v. four doses, for 2 weeks), but the patient remained intermittently feverish, necessitating the change to vancomycin (30 mg/kg/day i.v. two doses, for 4 weeks) based on the drug sensitivity test results. The patient was referred for surgical repair. The CAF was identified (Figure 1I), with complete resection vegetations on the orifice of the fistula in the RA. Fistula repair was performed with a Dacron patch inside the RA. During intraoperative exploration, no vegetations were found at the mitral or aortic valves. The patient tolerated the procedure well without complication. She uneventfully recovered following the operation. Postoperatively, she received intravenous antibiotics for 6 weeks. Antiplatelet therapy was recommended for 6 months. At 18 months follow-up, the patient was asymptomatic without complications.

## Discussion

CAF is a rare abnormal vascular connection between a coronary artery and any heart cavity or vessel. The most common sites of drainage are low-pressure structures, such as the right ventricle, RA, and pulmonary artery (5). LMCA aneurysm combined with LMCA fistulous connection to the RA is an extremely rare finding.

Most patients with CAF do not demonstrate any symptoms throughout their lives due to their small and insignificant left-to-right shunts. CAFs have different symptoms on the basis of their size and shunt flow (6). They may cause angina pectoris from myocardial ischemia due to coronary steal phenomenon, exertional dyspnea, syncope, and palpitations. The most important and dangerous complications of CAF include congestive heart failure, myocardial ischemia, thrombosis and embolism, pulmonary hypertension, aneurysm rupture, and endocarditis (7).

Echocardiography, CT, MRI, and coronary angiography are useful diagnostic modalities (8). In particular, CT has become an important noninvasive method for coronary artery anomalies, and it could clearly diagnose different types of CAF (9). CT angiography could clearly display the specific location, size, and morphology of the fistula, enabling surgeons to better understand the possible anatomical complexity of the CAF before surgery. Coronary angiography remains the gold standard in diagnosing CAF. It is used to determine the size, number, and anatomical characteristics of the fistulous tract, but it is expensive and invasive. In this case, multimodality imaging was used for diagnosis and procedural planning for CAF.

The patient had chest tightness and chest pain after exertion for 1 year. She also presented with intermittent fever of unknown origin for 1 month. Cardiac examination revealed a heart murmur, and blood cultures grew *S. sanguinis*. TTE found multiple vegetations in the heart. The patient was diagnosed with infective endocarditis on the basis of the modified Duke criteria and the ESC guidelines improvement from 2015 (10).

Symptomatic and asymptomatic patients at risk of complications in the future are recommended to receive treatment (2). No standardized treatment is currently available for CAF. The surgeon must decide the method of operation in accordance with the patient's condition and the type of CAF. The treatments include ligation, surgical patch closure, bypass graft, and transcatheter closure (11). This case underwent successful surgical repair after systematic anti-infection treatment.

In conclusion, infective endocarditis is an extremely rare but life-threatening complication of CAF. The 1-year mortality rate of infective endocarditis was around 30% in different series (12). The mortality rates could be reduced by strictly adhering to a standard therapeutic protocol.

## Data Availability

The original contributions presented in the study are included in the article/supplementary material, further inquiries can be directed to the corresponding author.
